# Peripheral T cell receptor beta immune repertoire is promptly reconstituted after acute myocardial infarction

**DOI:** 10.1186/s12967-019-1788-4

**Published:** 2019-02-06

**Authors:** Dan Li, Longgang Hu, Qing Liang, Cuijuan Zhang, Yunzhen Shi, Bin Wang, Kejia Wang

**Affiliations:** 1 Department of Cardiology, The Affiliated Hospital of Qingdao University, Qingdao, Shandong China; 2 College of Basic Medicine, Qingdao University, Qingdao, Shandong China; 3 Department of Cardiovascular Medicine, The Affiliated Cardiovascular Hospital of Qingdao University, Qingdao, China; 4 Center of Patients, West China Second University Hospital, Sichuan University, Chengdu, China

**Keywords:** T cell receptor beta, Immune repertoire, Acute myocardial infarction, Next-generation sequencing

## Abstract

**Background:**

Acute myocardial infarction (AMI) is characterized by an inflammatory process in which T cell plays a key role. However, the profile of immune microenvironment in AMI is still uncertain. High-throughput sequencing of T cell receptor (TCR) provides deep insight into monitoring the immune microenvironment.

**Methods:**

30 patients with AMI were enrolled and 30 healthy individuals were recruited as controls. Flow cytometer were used to analyze the distribution of αβ T cells and their CD69 expression from peripheral leukomonocytes. TCRβ repertoire library was amplified by two-round multiplex PCR and detected by next-generation sequencing (NGS).

**Results:**

The percentage of αβ T cells in AMI patients were significantly restricted than those in healthy controls, while the highly activated αβ T cells along with distinguishing usage of variable (V), diversity (D) and joining (J) gene segments were also found in AMI patients. In addition, AMI induced a significantly restricted CDR3 amino acid (AA) diversity and remarkably reconstituted TCR immune repertoires. Finally, we identified several AMI-associated tendency of CDR3 AAs expression after AMI.

**Conclusions:**

Our work suggests that the aberrant αβ T cells distribution and activation may associated with the pathogenesis of AMI and demonstrates a reconstitution of TCRβ immune repertoire after AMI.

**Electronic supplementary material:**

The online version of this article (10.1186/s12967-019-1788-4) contains supplementary material, which is available to authorized users.

## Background

Acute myocardial infarction (AMI), considered a lipid-related chronic inflammatory disease, often occurs on the basis of coronary atherosclerosis [1]. In general, AMI induces considerable remodelling of the myocardial tissue which leads to abnormal contractile function and heart failure, both constituting the prevailing cause for morbidity and mortality worldwide [2]. There is a global increase in the incidence of AMI in both male and female. It is estimated that AMI is responsible for over 8 million deaths each year, accounting for 5% of the global burden of disease [3].

Currently, it is widely accepted that T cells are the main immune competent cells meditated development of atherosclerotic plaques and local thrombosis [4]. It is believed that AMI is associated with T cell-associated cytokine imbalance that may act as predictors for ischemic heart disease outcomes [5, 6]. The myocardium is subject to myocardial ischaemia–reperfusion injury induced by re-establishment of blood flow though the majority of AMI patients is received prompt reperfusion. Emerging studies indicate that critical role of T cell in the pathogenesis of AMI injury and post myocardial infarction healing [7]. The balance of T cell-meditated immunity by the host is a major determinant of patient outcome [8], and overactive T cells, continually, lead to the progression of inflammatory response, resulting in organ dysfunction. Therefore, the potential immunosuppressive approaches may be used for the treatment of patients affected by AMI [8, 9]. Interestingly, the decrease of T lymphocyte count occurs in the circulatory system after AMI [10, 11]. The intrinsic mechanism between T cell response and AMI injury is still to need further exploration.

T cells can be divided into two major subsets characterized by the surface expression of a TCR α and β chain (αβ T cell) or γ and δ chain (γδ T cell). A majority of T cells in peripheral blood expresses is αβ T cells, while only 5–10% lymphocytes in peripheral blood are γδ T cells. T cells bear unique TCRs generated by random somatic recombination of V/(D)/J gene segments. T cell priming requires TCR ligation, by the cognate major histocompatibility complex (MHC) on antigen-presenting cells (APCs) to recognize antigens [12, 13]. It is considered that αβ T cells can sense external environmental trigger (viral or bacterium infection) and an endogenous stimulus (homeostasis) by TCR to mediate immunosurveillance and immunoregulation, and TCR will be reconstituted in response to stimuli (infection and disease) [14–16]. Nevertheless, the association of TCR repertoires in AMI has not been clarified as yet.

In the basis of recent literature and finding, we hypothesized that αβ T cells exert a critical function in the pathogenesis and pathophysiology of AMI, and an essential evolution of TCR repertoires may be found due to AMI-induced aberrant inflammatory microenvironment. In this study, we detected the count and activity of αβ T in peripheral blood, and NGS was used to monitor the expression pattern and clonality of TCR repertoire of αβ T cells in AMI patients. The manipulation of the heart-specific TCR immune repertoires can improve our basic understanding of T cell immunology and help to identify optimal TCRs for immunotherapy.

## Methods

### Participants

A total of 30 patients who diagnosed with AMI admitted to the Department of Cardiology, The Affiliated Hospital of Qingdao University were enrolled. The criteria for diagnosis of AMI patients were based on the third Universal Definition of Myocardial infarction: (1) Acute ischemic chest pain within 24 h; (2) Electrocardiogram change of acute myocardial infarction (pathological Q wave, ST-segment elevation or depression) and (3) Rise of cardiac biomarkers cTnI level [17]. The diagnosis was confirmed by coronary angiogram at the admission and all of AMI patients were received reperfusion by primary percutaneous intervention (PCI) concomitantly. Additionally, 30 healthy individuals without clinical sign of myocardial ischaemia were included as controls. This study was approved by the Ethics Committee of The Affiliated Hospital of Qingdao University and written informed consent was obtained from each participant.

### Lymphocytes isolation and flow cytometry analysis

Peripheral blood samples of patients with AMI was obtained within 24 h of the onset of symptoms, the blood of patients with controls were obtained immediately after hospital admission. The blood cell count was performed using Sysmex XN (Sysmex Corporation, Kobe, Japan) in the clinical laboratory of our hospital. Lymphocytes were isolated by using Ficoll (Solarbio Life Sciences, Beijing, China) density gradient centrifugation according to instruction. Subsequently, the isolated cells were washed twice with PBS for further experiments. To detect the αβ T cells, isolated lymphocytes were incubated with fluorescently conjugated antibodies directed against mouse CD3e (HIT3a), TCRα/β (IP26) and CD69 (FN50) from Biolegend (Beijing, China) for 20 min at room temperature in the dark. Cell counting was conducted using a BD Accuri C6 (BD Biosciences, Mountain View, CA, USA) and data was obtained and analyzed with BD Accuri C6 Software (BD).

### RNA extraction and TCR repertoires library preparation

RNAprep Pure Cell/Bacteria Kit (Tiangen Biotech, Beijing, China) was used to extract RNA from the isolated lymphocytes. The quality and quantity of RNA were measured by using a NanoDrop spectrophotometer (Thermo Fisher Scientific, USA). First-strand cDNA was synthesized using a Transcriptor First Strand cDNA Synthesis Kit (Roche Applied Science, Penzberg, Germany) according to the manufacturer’s protocol on a T100TM Thermal Cycler (Bio-Rad Inc., CA, USA). A total of 200 ng RNA was performed in the reverse transcription reaction. TCRβ repertoire library was prepared by two-round multiplex PCR using specific primers designed for functional V and C gene segments of TCRβ chain (Additional file 1: Fig. S1). TCRβ chain repertoire amplification was performed as described in our previous work [18]. The PCR products were loaded on 1% TBE-Agarose for gel electrophoresis and purified using the QIAquick Gel Extraction Kit (Qiagen). Illumina HiSeq X Ten platform was used for high-throughput sequencing of TCRβ chain repertoire.

### Data processing

Paired-end V, D and J sequences of TCRβ chain were identified using BLAST Plus on IMGT database (http://www.imgt.org/) by a standard algorithm after filtering the low-quality reads. Heatmaps and circular plots were created to reflect the frequencies of V, J gene segments and paired V–J combinations, respectively. Gini coefficient, Shannon diversity and Rank-abundance were used for assessing the richness and diversity of TCR as previously described [19–21].

### Statistical analysis

All statistical analyses were performed using GraphPad Prism 6.0 (GraphPad Software, La Jolla, CA) using a 2-tailed, unpaired Student’s t-test or Mann–Whitney U test was for two groups comparisons. Data are presented as the mean ± standard deviation. Significance was accepted at *P *< 0.05.

## Results

### Clinical characteristics of the subjects

**Fig. 1:**
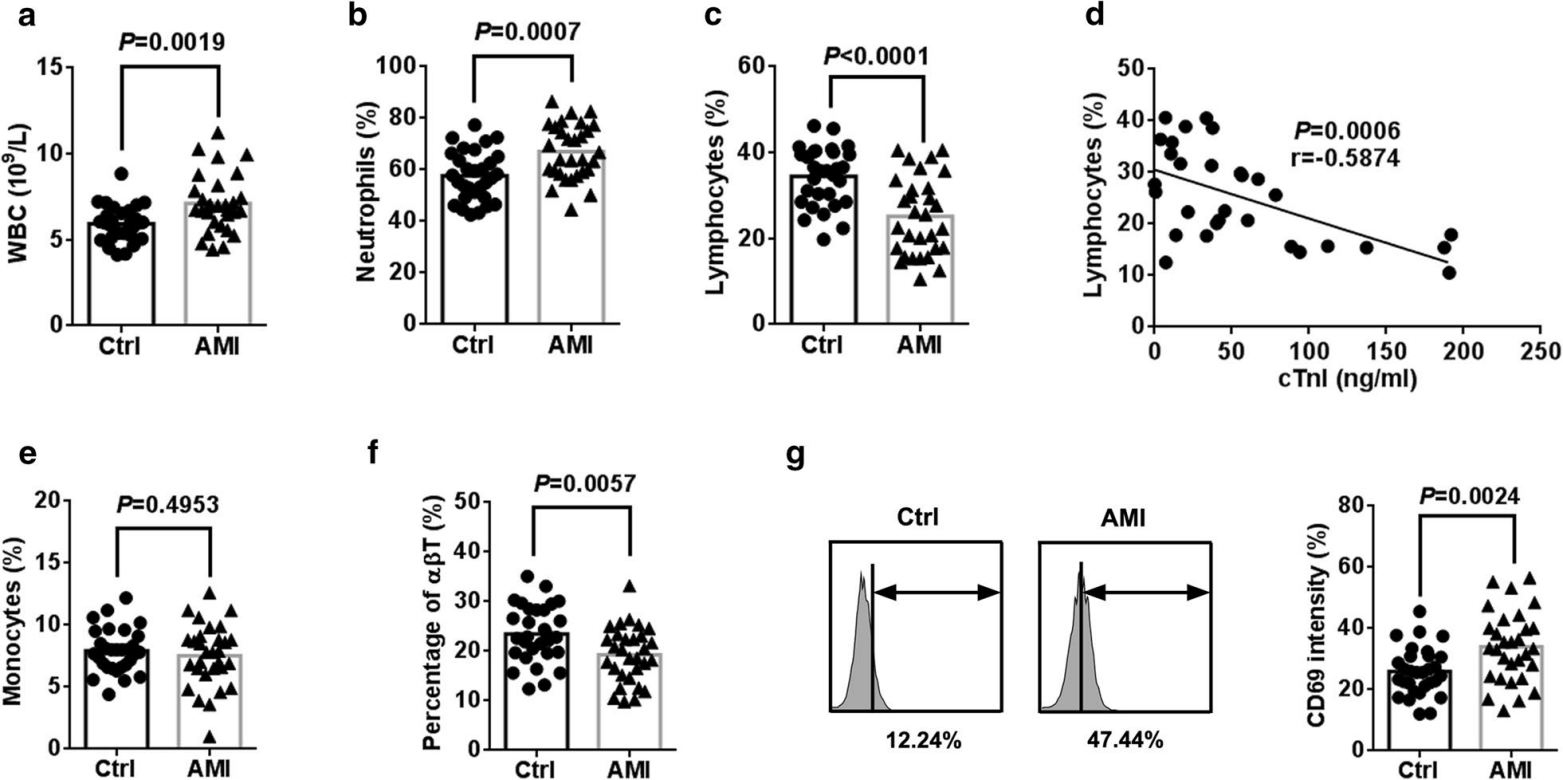
AMI leads to αβ T cells activation. Peripheral blood samples were obtained from 30 healthy controls and 30 AMI patients. **a** The count of WBC was measured from peripheral blood samples. The percentage of neutrophils (**b**), lymphocytes (**c**), monocytes (**e**) was detected by Sysmex XN in our clinical laboratory. **d** The correlation between cTnI level and lymphocytes was calculated in AMI patients. Spearman’s correlation coefficient r = − 0.5874, *P* = 0.0006 (n = 30). **f** The percentage of αβ T cells from peripheral blood was analyzed by flow cytometry. **g** αβ T cells were gated and tested for expression of CD69

Demographic characteristics and baseline clinical features of participants including gender, age, past medical history and biochemical data were summarized in Table 1. No significant differences were observed with gender, age, hypertension, diabetes mellitus, total cholesterol and history of smoking between two groups. The concentration of cTnI was significantly higher in AMI patients than that in healthy controls (Table 1). We also found that levels of peripheral WBC and neutrophils were significantly elevated in AMI patients (Fig. 1a, b). Interestingly, the percentage of lymphocytes was significantly decreased in AMI patients compared to controls (Fig. 1c), and the concentration of cTnI was negatively associated with lymphocytes levels (Fig. 1d). No significant variation in monocytes levels was verified in AMI patients (Fig. 1e).

**Table 1:** Baseline characteristics of the enrolled subjects

	Healthy controls (n = 30)	AMI patients (n = 30)	*P* value
Age (year)	57.6 ± 11.6	58.8 ± 10.4	NA
Gender (male/female)	17/13	18/12	NA
Arterial hypertension (%)	30%	40%	NA
Diabetes mellitus (%)	20%	30%	NA
Smoking (%)	23.3%	30%	NA
History of AMI (%)	0	0	NA
BMI (kg/m^2^)	25.8 ± 2.2	26.4 ± 3.3	NA
Total cholesterol (mmol/L)	5.04 ± 1.28	5.12 ± 1.24	NA
cTnI (ng/mL)	56.8 ± 56.5	0.012 ± 0.040	< 0.001

*BMI* body mass index, *cTnI* cardiac troponin-I

### AMI activates αβT cells in AMI patients

To explore the status of αβ T cells in response to AMI, we measured the population of αβ T cells from peripheral blood by using flow cytometer. As expected, a lower percentage of peripheral αβ T cells were observed in AMI patients compared to healthy controls (Fig. 1f). To determine whether AMI activates αβ T cells, we also examined the expression of CD69 of αβ T cells in peripheral blood. Strikingly, there was, compared to controls, obvious increase in CD69 level of αβ T cells, indicating that a significant activation of peripheral αβ T cells occurred in AMI patients (Fig. 1g).

### Profiling of V, D and J gene segments usage after AMI

**Fig. 2:**
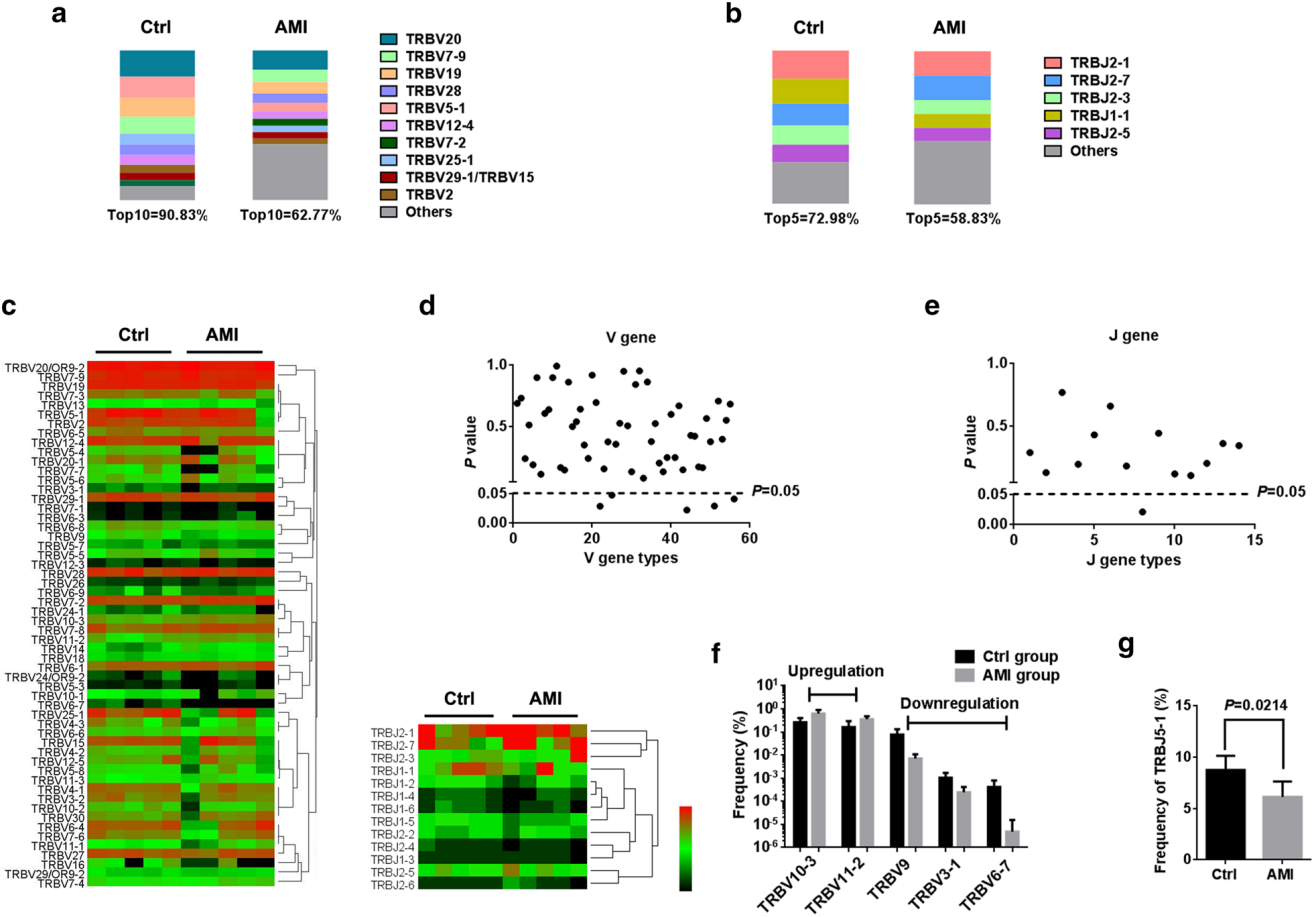
The usage patterns of V and J gene segments after AMI. High-throughput sequencing of 10 PCR products (five controls and five AMI patients) amplified from peripheral lymphocytes for TCRβ repertoire analysis. The distribution of Top10V (**a**) and Top5J (**b**) genes expression. Colors indicate Top10/Top5 clones (each color represents a clone); grays represent non-Top10/Top5 clones. **c** Heatmaps of hierarchical clustering of V and J gene segments frequencies. The frequencies of V (**d**, **f**) and J (**e**, **g**) gene segments usage that had significant differences between healthy controls and AMI patients

Next, the usages patterns of V, D and J gene were measured by NGS approach based on multiplex PCR from peripheral αβ T cells. This yielded on 5.13 × 10^6^ to 11.11 × 10^6^ productively TCRβ blast reads per sample. The total number of TCRβ CDR3 reads was 0.57 × 10^4^ to 7.60 × 10^4^, with an average of 3.70 × 10^4^ CDR3 clonotypes per sample (Additional file 2: Table S1). In addition, a total of 56 distinct V gene segments and 14 distinct J gene segments from all samples (Additional file 3: Table S2). Overall, the usages of V and J gene segments were dominated by high-frequency segments in healthy controls, which top10 TRBV accounted for 90.83% and top5 TRBJ accounted for 72.98%. In contrast, top10 TRBV only accounted for 62.77% and top5 TRBJ accounted for 58.83% in AMI patients (Fig. 2a, b). The most frequent V gene segments were TRBV20 (17.51% in healthy controls, 12.77% in AMI patients). The most frequent J gene segments were TRBJ2-1 (18.43% in healthy controls, 15.95% in AMI patients). Generally, the usage patterns and frequencies of most V and J genes were similar between healthy controls and AMI patients (Fig. 2c–e). However, these were significant differences in frequency of TRBV10-3, TRBV11-2, TRBV9, TRBV3-1, TRBV6-7 and TRBJ5-1 (Fig. 2f, g).

### Lower V–J and V–D–J combinations in AMI patients

**Fig. 3:**
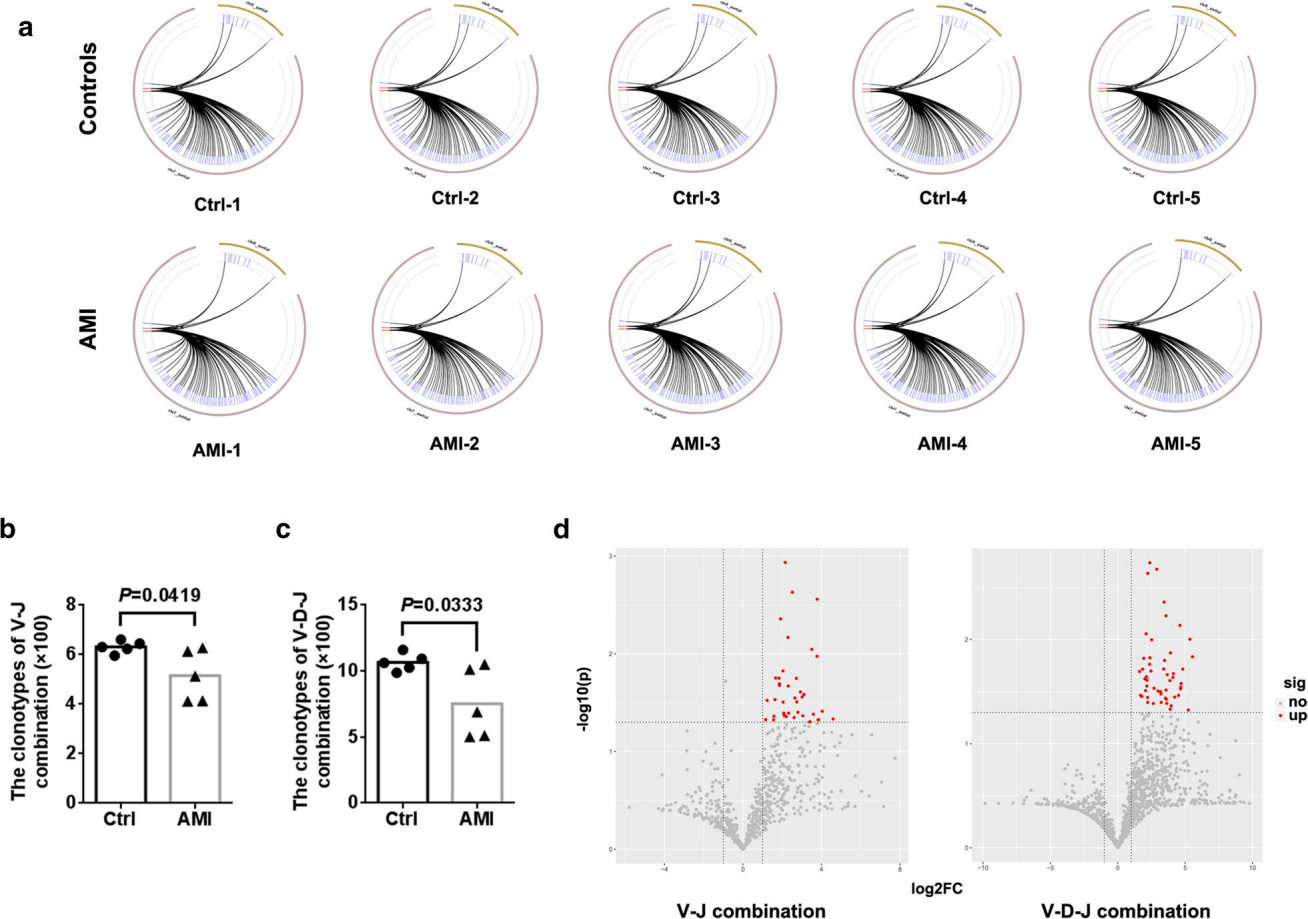
The usage patterns of V–J and V–D–J combination. **a** Circular plots representing TCRβ loci were identified from αβ T cells. The clonotypes of V–J (**b**) and V–D–J (**c**) combinations were counted from sequencing data. **d** Volcano plots showing the comparison of V–J and V-D–J combinations between healthy controls and AMI patients (red dots refer to the differentially expressed combinations with statistical significance)

Furthermore, we also analyzed the composition of paired V–J combinations and paired V–D–J combinations (Additional file 4: Table S3, Additional file 5: Table S4). Analysis of high-throughput sequencing of TCRβ chain repertoire led to the identification of 708 distinct V–J combinations and 1304 distinct V–D–J combinations (Fig. 3a). Of note, AMI significantly resulted in reduced the clonotypes of V–J and V–D–J combinations (Fig. 3b, c). The volcano plots were generated according to the usage frequency of V–J combinations and V–D–J combinations. Compared to controls, there were 39 V–J combinations and 51 V–D–J combinations that exhibited significantly abnormal usage in AMI patients. Interestingly, the frequencies of all altered V–J and V–D–J combinations were elevated in AMI patients, compared to healthy controls (Fig. 3d).

### AMI induces a diminished diversity of CDR3 AA

**Fig. 4:**
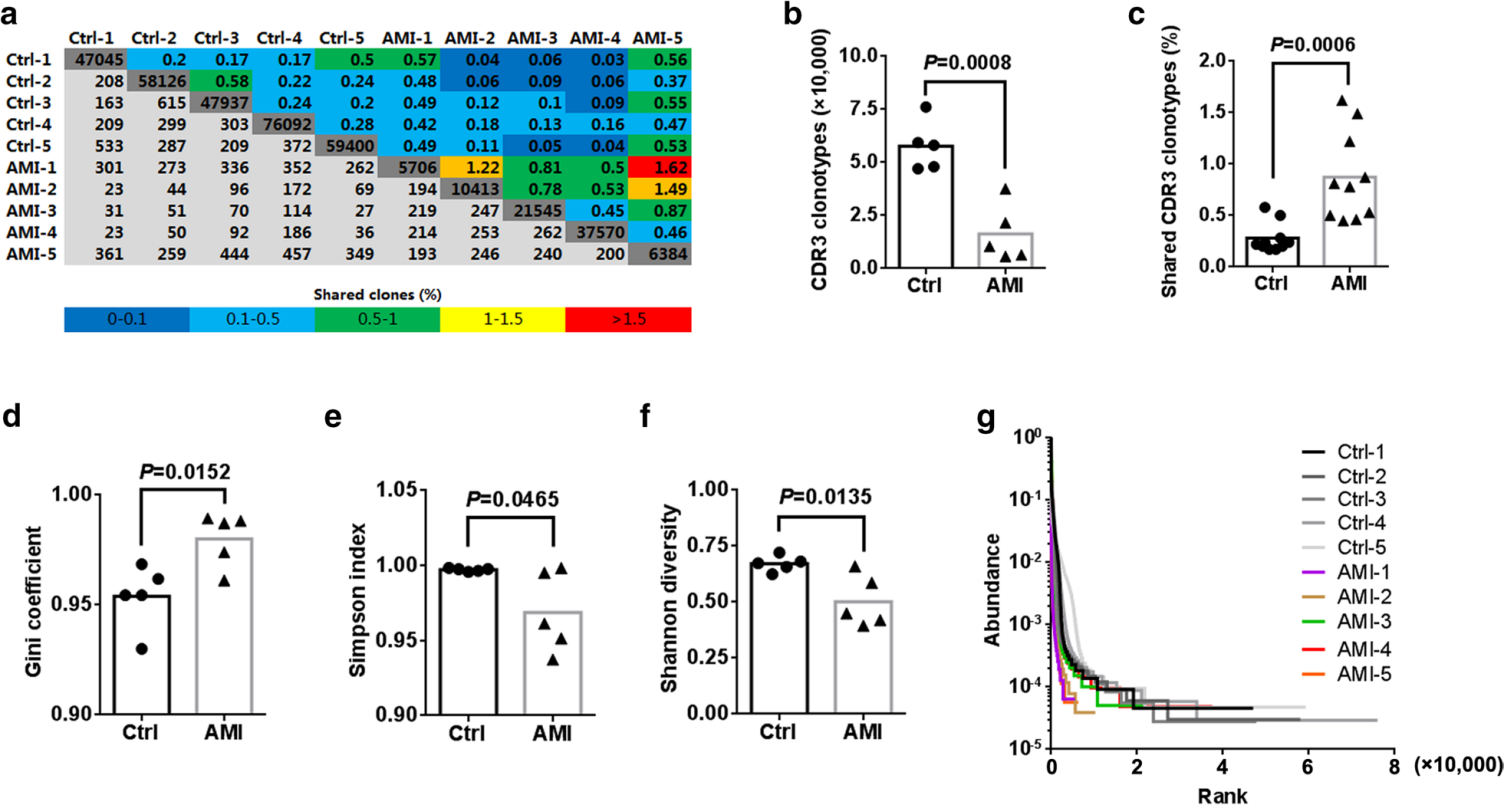
The diversity of TCRβ CDR3 AA clonotypes. **a** Quantification (plot grays) and frequencies (plot colors) of overlapping TCRβ clonotypes. Light grays indicate the overlapping clonotypes, while dark grays indicate the total clonotypes per sample. Comparison of TCRβ repertoire diversity by total CDR3 AA clonotypes (**b**), shared CDR3 AA clonotypes (**c**), Gini coefficient (**d**), Simpson index (**e**) and Shannon index (**f**) between healthy controls and AMI patients. **g** Rank-Abundance analysis of CDR3 AA clonotypes among different samples

Types of CDR3 AA clones determine the diversity of the TCR repertoires. In the current study, 360,274 distinct CDR3 AA clonotypes was identified (Additional file 6: Table S5). Although total TCR CDR3 AA clones were distinguishing among different individual, AMI, of note, led to obvious decreases in TCRβ base sequence clonotypes and CDR3 AA clonotypes (Additional file 1: Fig. S2a, b and Fig. 4a, b). Next, we quantified overlap of TCRβ base sequence and CDR3 AA clonotypes among different sample and found that overlap of TCRβ base sequence and CDR3 AA clonotypes in AMI patients were substantially higher than it in controls (Additional file 1: Fig. S2a, c and Fig. 4a, c). Gini coefficient, Simpson index and Shannon diversity suggested remarkably lessened CDR3 AA diversity in AMI patients (Fig. 4d–f). Rank-abundance analysis also exhibited a shrunken CDR3 AA richness and evenness in AMI patients (Fig. 4g).

### Resetting of the CDR3 AA in AMI patients

**Fig. 5:**
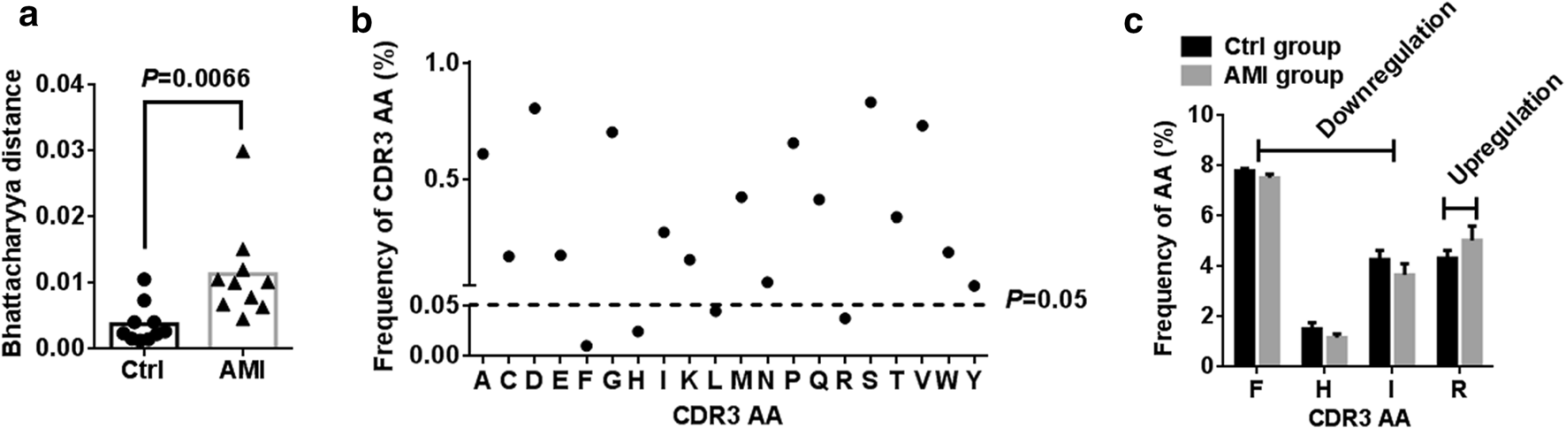
Reconstitution of CDR3 AA after AMI. **a** Bhattacharyya distance analysis for the similarity of CDR3 AA from different group. **b** Comparison of the fraction of Top20 CDR3 AAs between healthy controls and AMI patients. **c** The frequencies of CDR3 AA that had significant differences were shown

In order to investigate the reconstitution of CDR3 AA, Bhattacharyya distance was used to assess the similarity. As expected, Bhattacharyya distance clearly demonstrated a high similarity of CDR3 AA in AMI patients compared to healthy controls (Fig. 5a). In addition, we evaluated the frequency of CDR3 AA usage in different samples (Additional file 7: Table S6). Of note, comparison of CDR3 AA usage showed that AMI induced aberrant CDR3 AAs usage of phenylalanine (downregulation), histidine (downregulation), leucine (downregulation) and arginine (upregulation) (Fig. 5b, c). However, there were no significant differences observed in length tendency in CDR3 AA length after AMI (Additional file 1: Fig. S3).

## Discussion

Aberrant T cell function caused by AMI usually results in acute and chronic inflammatory processes that impair cardiac function [7, 22, 23]. Currently, despite a profound understanding of the adaptive immune system, and specifically the T cell response, in AMI, much less is known about TCR rearrangement. Accumulating evidence demonstrate that TCR repertoires will be rapidly reconstituted in response to endogenous and exogenous stimuli for immunosurveillance and immunoregulation [24–26]. Our preceding study indicates that TCR acts more like a “commander” than like an “executor” to monitor immune microenvironment [18]. Therefore, identifying and tracking TCR immune repertoires provide a novel method to understand the association between T lymphocytes and AMI injury.

The vast majority of data support that patients suffering AMI have significantly increased peripheral blood T cell activation though the decrease of T lymphocyte count occurred in the circulatory system [10, 11, 27, 28]. Following AMI, CD4^+^ T cells can be activated, by presentation of myocardial peptides by dendritic cell [29]. It is considered that this activation process occurs within days and requires an intact T-cell receptor repertoire. Recognition of cardiac autoantigens by TCR, presumably, is critical for maintaining the balance of control of AMI injury vs. T cell response, and facilitate wound healing of the myocardium [29]. Consistent with previous studies, our results revealed reduced percentage and enhanced activation of αβ T cells in peripheral blood after AMI, demonstrating the crucial role of αβ T cells in AMI [11, 30]. Surely, enhanced activation of αβ T cells can be attributed to AMI injury and heart failure. Nevertheless, the cause of diminishing αβ T cells in the process of AMI is still uncertain. It is speculated that several T cell subpopulations which exert immunosuppressive effect on myocardial inflammation vanish in this course, and TCR immune repertoires rearrangement occurs in order to recognize cardiac autoantigens, resulting T cells activation and myocardium injury. In this course, a massive activated T cells itself provides exact identification synergistically with other immunocytes to promote the pathogenesis.

Our work revealed an intact TCRβ profile of V, D and J genes usage and the distribution of CDR3 AA clonotypes after AMI. In the current study, the frequencies of V and J genes exhibited more centralized distribution though the majority of V and J genes were similar between healthy controls and AMI patients. It is noteworthy that the high clonal expansion frequencies of the TRBV10-3, TRBV11-2 and the low clonal expansion frequencies of the TRBV9, TRBV3-1, TRBV6-7 and TRBJ5-1 were determined in AMI patients. It is suggested that such expand TRBV clones may be reactive T cell clones directed against AMI, while such lessened TRBV/TRBJ clones may act as immunosuppressive T cell clones in AMI. Moreover, AMI induced a diminished composition of V–J/V–D–J combination. Interestingly, the frequencies of all V–J/V–D–J combinations that had significant differences after AMI were upregulated unanimously, partly as a result of reduced composition of V–J/V–D–J combination and partly because AMI restrains immunosuppressive T cell subsets emergence. These data were consistent with the results in Fig. 1, which indicate that a lower percentage of peripheral αβ T cells was observed in AMI patients.

CDR3, composed of variable (V), diversity (D), and joining (J) domains from the terminal of the V domain to the beginning of the J domain, is considered as a critical region to recognize antigen. The specificity and diversity of TCR repertoires is dependent of CDR3 AA clonotypes [31, 32]. In this work, obviously decreased CDR3 clonotypes and altered CDR3 AAs were observed in AMI patients, suggesting that AMI led to a shrunken TCRβ diversity and VDJ recomposition. Among AMI patients, the proportion of shared CDR3 AA was obviously elevated compared to that among healthy controls, which imply that AMI induced the usage of several key high-frequency CDR3 AAs. Our observation of transformation of TCRβ repertoire after AMI provides evidence of AMI-associated immune repertoire in AMI injury progression. These results indicate that transformation of TCR immune repertoires is indispensable for antigen recognition in the adaptive immune system for myocardial injury.

Undoubtedly, AMI-responsive TCRβ immune repertoire represents a “footprint” of immune microenvironment in peripheral blood, which will be conducive to better understanding of the function of αβ T cell in myocardial injury after AMI. However, the mechanism and effect of TCRβ reconstitution in T cell activation are still not clear, and how TCRβ immune repertoire modulates the inflammatory milieu needs to be further investigated. Of note, a recent study finds the difference in expression pattern and clonality of TCR γδ T cells between AMI patients and healthy individuals, reflected by the obviously restricted TCR γδ subfamilies expression in γδ T cells from AMI patients [33]. The preceding data along with our result indicate an important role of αβ and γδ T cells in AMI injury, and reconstitution of TCR immune repertoires participate in modulation of inflammatory status.

## Conclusions

Our work first reveals the characteristic of the TCRβ immune repertoire after AMI. Through its tracking of the kinetics of the reconstitution of TCRβ, we find significantly restricted TCRβ immune repertoire and the discrepancy in the expression of αβ T subsets, which may be related to the immune response and clinical outcome. These data provide a novel insight to identify signatures of AMI-associated immune microcircumstance, which will accelerate discoveries of novel immunotherapy for AMI patients.

## Additional files

**Additional file 1: Fig. S1.** Two-round nested amplicon arm-PCR for TCRβ immune repertoire. n = 5 per group. **Fig. S2.** The clonotypes analysis of TCRβ base sequence. **a** Quantification (plot grays) and frequencies (plot colors) of base sequence clonotypes. Light grays indicate the overlapping clonotypes, while dark grays indicate the total clonotypes per sample. **b** Comparison of total clonotypes of TCRβ base sequence. **c** The percentage of overlapping base sequence between healthy controls and AMI patients. **Fig. S3.** The distribution of CDR3 AA length between healthy controls and AMI patients. n = 5 per group.

**Additional file 2: Table S1.** The analysis of TCRβ immune repertoire sequencing data.

**Additional file 3: Table S2.** The distribution of V and J gene segments in each sample.

**Additional file 4: Table S3.** The distribution of V-J combinations in each sample.

**Additional file 5: Table S4.** The distribution of V-D-J combinations in each sample.

**Additional file 6: Table S5.** The distribution of CDR3 AA clonotypes in each sample.

**Additional file 7: Table S6.** The distribution of AA usage in CDR3.

## Abbreviations

AMI: acute myocardial infarction
TCR: T cell receptor
CDR3: complementary determined regions 3
AA: amino acids
MHC: major histocompatibility complex
APCs: antigen-presenting cells
NSG: next-generation sequencing
cTnI: cardiac troponin-I
PCI: primary percutaneous intervention
TRBV: TCRβ V gene
TRBJ: TCRβ J gene
IMGT: ImMunoGeneTics
