# Supplementary material for: Peripheral T cell receptor beta immune repertoire is promptly reconstituted after acute myocardial infarction
Source: J Transl Med. 2019 Feb 6;17:40. doi: 10.1186/s12967-019-1788-4 (PMC6366076; doi:10.1186/s12967-019-1788-4)
Supplement: Supplementary file 1 — Additional file 1: Fig. S1. Two-round nested amplicon arm-PCR for TCRβ immune repertoire. n = 5 per group. Fig. S2. The clonotypes analysis of TCRβ base sequence. a Quantification (plot grays) and frequencies (plot colors) of base sequence clonotypes. Light grays indicate the overlapping clonotypes, while dark grays indicate the total clonotypes per sample. b Comparison of total clonotypes of TCRβ base sequence. c The percentage of overlapping base sequence between healthy controls and AMI patients. Fig. S3. The distribution of CDR3 AA length between healthy controls and AMI patients. n = 5 per group. [file 12967_2019_1788_MOESM1_ESM.doc]

**Peripheral T cell receptor beta immune repertoire is promptly reconstituted after acute myocardial infarction**

Dan Li^1^, Longgang Hu^3^, Qing Liang^2^, Cuijuan Zhang^1^, Yunzhen Shi^4^, Bin Wang^2^, Kejia Wang^2^

^1^ Department of Cardiology, The Affiliated Hospital of Qingdao University, Qingdao University, Qingdao, Shandong, China

^2^ College of Basic Medicine, Qingdao University, Qingdao, Shandong, China

^3^ Department of Cardiovascular Medicine, The Affiliated Cardiovascular Hospital of Qingdao University, Qingdao, China

^4^ Center of Patients, West China Second University Hospital, Sichuan University, Chengdu, China

Correspondence should be addressed to Kejia Wang (Email: princewkj@qdu.edu.cn)


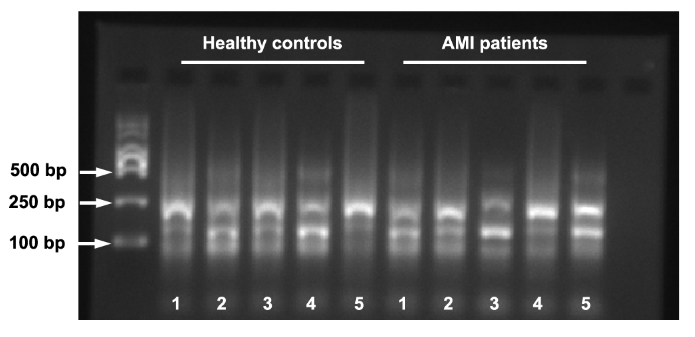
**Fig. S1** Two-round nested amplicon arm-PCR for TCRβ immune repertoire. n = 5 per group


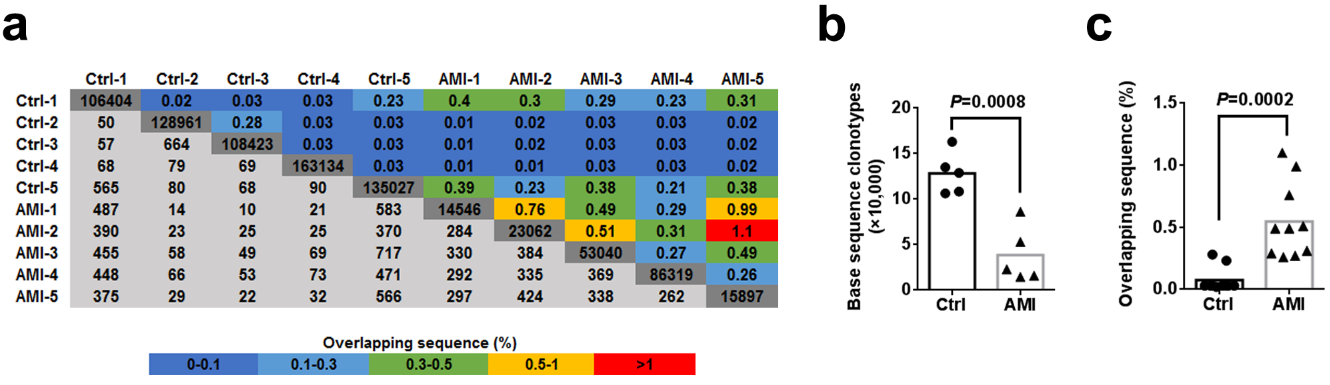


**Fig. S2** The clonotypes analysis of TCRβ base sequence. **a** Quantification (plot grays) and frequencies (plot colors) of base sequence clonotypes. Light grays indicate the overlapping clonotypes, while dark grays indicate the total clonotypes per sample. **b** Comparison of total clonotypes of TCRβ base sequence. **c** The percentage of overlapping base sequence between healthy controls and AMI patients.


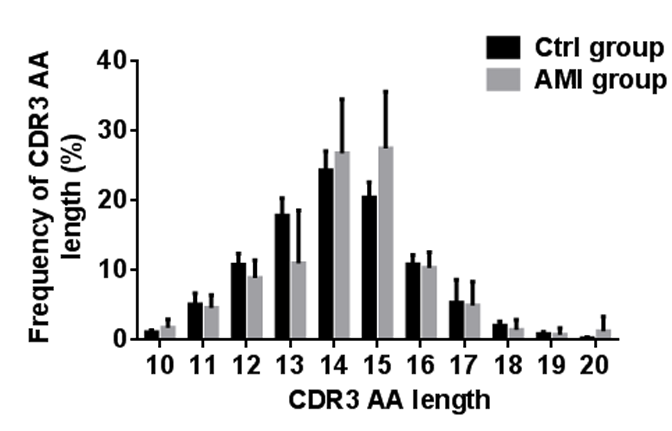
**Fig. S3** The distribution of CDR3 AA length between healthy controls and AMI patients. n = 5 per group
